# Supplementary material for: Modulation of α-synuclein aggregation amid diverse environmental perturbation
Source: eLife. 2024 Aug 1;13:RP95180. doi: 10.7554/eLife.95180 (PMC11293868; doi:10.7554/eLife.95180)
Supplement: Figure 8—source data 1. [file elife-95180-fig8-data1.docx]

Figure 8-source data 1: Shannon entropy^[50]^ for various datasets and αS

| **Dataset** | **min** | **mean** | **max** |
| --- | --- | --- | --- |
| LLPS+ | 2.08 | 3.76 | 4.19 |
| LLPS- | 2.03 | 3.75 | 4.17 |
| PDB* | 3.08 | 4.00 | 4.24 |
| αS | —- | 3.65 | —- |
