# Supplementary material for: Modulation of α-synuclein aggregation amid diverse environmental perturbation
Source: eLife. 2024 Aug 1;13:RP95180. doi: 10.7554/eLife.95180 (PMC11293868; doi:10.7554/eLife.95180)
Supplement: Figure 8—source data 2. [file elife-95180-fig8-data2.docx]

Figure 8-source data 2: normalized Kyte Doolittle hydrophobicity^[51]^ scores for various datasets and αS

| **Dataset** | **min** | **mean** | **max** |
| --- | --- | --- | --- |
| LLPS+ | -2.19 | -0.75 | 0.95 |
| LLPS- | -2.16 | -0.78 | 1.05 |
| PDB* | -1.50 | -0.21 | 1.59 |
| αS | —- | -0.41 | —- |
