# Supplementary material for: Modulation of α-synuclein aggregation amid diverse environmental perturbation
Source: eLife. 2024 Aug 1;13:RP95180. doi: 10.7554/eLife.95180 (PMC11293868; doi:10.7554/eLife.95180)
Supplement: Figure 8—source data 3. [file elife-95180-fig8-data3.docx]

Figure 8-source data 3: PLAAC NLLR^[52]^ scores for various datasets and αS

| **Dataset** | **min** | **mean** | **max** |
| --- | --- | --- | --- |
| LLPS+ | -0.558 | -0.206 | 0.936 |
| LLPS- | -0.650 | -0.035 | 0.936 |
| PDB* | -0.966 | -0.384 | 0.052 |
| αS | —- | -0.41 | —- |
