# Supplementary material for: Modulation of α-synuclein aggregation amid diverse environmental perturbation
Source: eLife. 2024 Aug 1;13:RP95180. doi: 10.7554/eLife.95180 (PMC11293868; doi:10.7554/eLife.95180)
Supplement: Figure 8—source data 4. [file elife-95180-fig8-data4.docx]

Figure 8-source data 4: catGRANULE^[53]^ scores for various datasets and αS

| **Dataset** | **min** | **mean** | **max** |
| --- | --- | --- | --- |
| LLPS+ | -1.37 | 2.37 | 9.83 |
| LLPS- | -2.03 | 1.43 | 8.26 |
| PDB* | -4.06 | 0.01 | 2.41 |
| αS | —- | 1.13 | —- |
