# Supplementary material for: Modulation of α-synuclein aggregation amid diverse environmental perturbation
Source: eLife. 2024 Aug 1;13:RP95180. doi: 10.7554/eLife.95180 (PMC11293868; doi:10.7554/eLife.95180)
Supplement: Figure 8—source data 5. [file elife-95180-fig8-data5.docx]

Figure 8-source data 5: Comparison of primary sequence derived features for various datasets and αS

| **Dataset** | **S** | **normalized hydrophobicity** | **NLLR** | **catGRANULE** |
| --- | --- | --- | --- | --- |
| LLPS+ | 3.76 | -0.75 | 0.206 | 2.37 |
| LLPS- | 3.75 | -0.78 | -0.035 | 1.43 |
| PDB* | 4.00 | -0.21 | -0.384 | 0.01 |
| αS | 3.65 | -0.41 | -0.438 | 1.13 |
